# The Impact of Learning From Failure on New Ventures’ Sustainable Development

**DOI:** 10.3389/fpsyg.2021.784518

**Published:** 2021-11-30

**Authors:** Ekaterina Shirshitskaia, Xue Zhou, Ling Zhang

**Affiliations:** Business School, Qingdao University, Qingdao, China

**Keywords:** entrepreneurial dynamic capability, strategic decision comprehensiveness, learning from failure, new ventures’ sustainable development, resource orchestration theory

## Abstract

How to absorb failure experiences to achieve reunification and turn crises into opportunities is crucial for enterprises. We examine the effect of learning from failure on new ventures’ sustainable development from the lens of resource orchestration theory. With 193 samples of entrepreneurs in Mainland China, this study provides the first quantitative evidence regarding how learning from failure influences new ventures’ sustainable development through entrepreneurial dynamic capability and strategic decision comprehensiveness. Stepwise regression analysis results show that learning from failure has a positive impact on the entrepreneurial dynamic capability and strategic decision comprehensiveness. Entrepreneurial dynamic capability and strategic decision comprehensiveness positively influence new ventures’ sustainable development, and exert mediating roles between learning from failure and new ventures’ sustainable development.

## Introduction

New ventures play a vital role in promoting the regional economy and solving the employment of residents. Therefore, supporting new ventures and establishing a competitive advantage for new ventures can help to promote business development as well as benefit the country’s livelihood (Yu and Pu, 2018). The Chinese government has introduced several policies to support the development of new ventures, such as loan support and preferential taxation, which have effectively promoted the development of new ventures. However, in the frequently changing business environment, most new ventures still face a variety of difficulties caused by internal factors or external factors of the environment, such as insufficient working capital, shortage of innovation resources, rising operating costs, and other survival difficulties. These development dilemmas make new ventures often suffer technical or management failures such as research failures, new product promotion failures, and strategic transformation failures. As an example of uncertain business environment, the epidemic swept the world in 2020, and all enterprises, especially new ventures, are experiencing unprecedented challenges, with international companies such as Yelp taking significant pay cuts and layoffs, Best Buy suspending more than 50,000 people, with endless information about enterprises in business difficulties. New ventures with newly created weaknesses are struggling and may fall apart if they are not careful. In the face of the tense and complicated economic situation at home and abroad, how to turn the crisis into peace, or even turn the crisis into opportunity has become an important topic of discussion between the academic and business sectors. For this reason, doing a good job in failure management and learning to discover the positive effects of failure is the key to new ventures to improve their survival and achieve sustainable development (Liu et al., 2019).

The epidemic in 2013 are similar to today’s situation, and the crisis response initiatives of enterprises in those years have certain significance for today’s enterprises, which can help them to get out of today’s difficulties. Therefore, we need to pay attention to the lessons learned from the past, both for the economy as a whole and for independent business organizations. When companies encounter failures and challenges, they must not only inspire and overcome difficulties together but also look to the future and establish a procedural governance mechanism. Specifically, the company would better form a corporate culture that correctly recognizes failure and provides guidance for actions after failure, such as holding a meeting to review the failure process and discuss the reasons for the failure. Companies need to recognize the fact of failure, summarize the experience of failure, learn from failure, and build new capabilities, new strategies, and new advantages based on past failures to lay the foundation for sustainable development. Previous studies have pointed out that entrepreneurial learning can support enterprises to update their knowledge and thus establish competitive advantages (Zollo and Winter, 2002). Based on the resource-based view, a stream of work notes that competitive advantage of enterprises comes from the appropriation and utilization of heterogeneous resources (Miller, 2010). Following this view, scholars then put forward the concept of dynamic capabilities, arguing that enterprises achieve resonance with the market through the reasonable allocation of heterogeneous resources and management capabilities to build up competitive advantage (Teece, 2010; Li et al., 2019). However, Renko et al. (2015) pointed out that the dynamic capabilities proposed based on incumbent firms did not significantly affect the competitive advantage of new ventures. Taken together, these literature streams suggest that impact of learning may act as resource allocation mechanisms that influence corporate sustainable development when there is a lack of clarify on the impact of learning from failure on new ventures’ sustainable development.

The resource orchestration theory has been a preeminent theory in explaining new ventures’ sustainable development (Miller, 2010). The resource orchestration theory suggests that new ventures’ sustainable development originates from the identification, acquisition, and use of resources based on subjective cognition by entrepreneurs (Miller, 2010). Consistent with this theory, learning from failure behavior as a way for entrepreneurs to adjust their cognition is impactful on the new ventures’ sustainable development, and its effectiveness is reflected through the entrepreneur’s resource identification and application process. According to the resource orchestration theory, the source of sustainable development lies in the satisfaction of customer value needs. Specifically, new ventures’ sustainable development relies on building resource portfolio, bundling resources to form capacity and allocate resources to form a strategic deployment. First, building a resource portfolio means acquiring external resources, accumulating internal resources, and stripping away non-core resources. Second, bundling resources to form capacity means developing and using resources to form stable, rich, and creative enterprise capacity. Third, to give full play to the allocation capability is to mobilize and allocate resources to form a strategic deployment (Wiklund and Shepherd, 2003; Sirmon et al., 2011). Therefore, realizing the sustainable development of enterprises through resource orchestration needs to be promoted from two aspects including capacity cultivation and strategic deployment (Rae and Carswell, 2001; Sun et al., 2020).

To begin narrowing the gap between what we know and what we need to know concerning how learning from failure influence new ventures’ sustainable development, two important theoretical lenses that allows us to bridge the gap according to resource orchestration theory are entrepreneurial dynamic capabilities and strategic decision comprehensiveness (Sirmon et al., 2007). Entrepreneurial dynamic capabilities highlight the characteristics of the integrated development of opportunity resources of entrepreneurial enterprises (Ardichvili et al., 2003; Corner and Wu, 2012). Strategic decision comprehensiveness as a reflection of the strategic deployment reflects the quality of the entrepreneur’s strategic decisions (Atuahene-Gima and Li, 2004). Specifically, we seek to answer the research question: to what extent, if any, do learning from failure influence new ventures’ sustainable development through entrepreneurial dynamic capabilities and strategic decision comprehensiveness?

Our work provides two important implications for several literatures. First, we confirm the logic of resource orchestration theory from an empirical view and enrich the research based on the resource orchestration theory. Failure learning emphasizes entrepreneur cognition, which is the change in cognition and strategy of entrepreneurs after experiencing failure situations or events, such as forming the coping logic of entrepreneurial activities. Based on the logical framework of cognition-behavior-performance, this study points out that the impact of failure learning on the sustainable development of entrepreneurship is that entrepreneurs build resource portfolios based on adjusted cognition and bundle resources to form entrepreneurial dynamic capabilities or leveraged configuration capabilities to improve strategic decision comprehensiveness. Second, by linking failure learning to new ventures’ sustainable development, this study enriches the literature on how the failure learning can help new ventures to improve their business success. Given the resource constraints faced by the founders or managers of new ventures, our research results provide practical information to help new ventures to get sustainable development with the guidance of the knowledge gained from previous failure experiences.

## Theoretical Foundation

### Learning From Failure

Entrepreneurship research defines failure into three categories. One is to equate failure with corporate failure (Watson and Everett, 1993). Since this definition includes the case where entrepreneurs take the initiative to shut down companies (Bates, 2002), they are often criticized by academia (Politis and Gabrielsson, 2009). The second is to define failure as a business failure, that is, a scenario where a startup company is forced to shut down because it cannot achieve its goals or repay its debts (Ucbasaran et al., 2009). The third type of failure is defined as a staged situation or fact that a start-up fails to achieve its expected goals in the process of creating or managing the enterprise (Politis and Gabrielsson, 2009). Entrepreneurship is full of uncertainty, and the entrepreneurial process is full of setbacks and obstacles. We adopt the third definition because it is more suitable for describing the theory and practice of entrepreneurship (Politis and Gabrielsson, 2009).

Entrepreneurial failure in this paper is a scenario in which the practical outcome of the entrepreneurial process deviates from the expected goal, such as the failure of financing for science and technology new ventures and the failure of new product development (Atuahene-Gima and Li, 2004; Cannon and Edmondson, 2010; Eggers and Song, 2015; Yamakawa et al., 2015). Cope (2005) first pointed out that entrepreneurial failure learning is one of the important directions of entrepreneurial learning research. Integrating the views of Cannon and Edmondson (2010) and Cope (2011), learning from failure is the process of reflecting and gaining lessons based on the failure experience to achieve strategy adjustment and value revision (Cannon and Edmondson, 2010; Cope, 2011). Therefore, learning from failure includes both cognitive revision and strategy change, with cognitive revision emphasizing the adjustment of cognitive biases and the identification of corporate strengths and weaknesses and strategy change emphasizing the improvement of management behaviors or operational processes (Cannon and Edmondson, 2010). On this basis, many studies have explored the impact of learning from failure on corporate performance, pointing out that learning from failure can enable companies to “learn from their mistakes” and reflect the wisdom dividends in performance outcomes (Atuahene-Gima and Li, 2004; Eggers and Song, 2015; Yamakawa et al., 2015; Boso et al., 2018). However, most of the existing studies draw on psychological theories to explore the internal and external changes brought about by entrepreneurial learning from failure from an emotional perspective, but there is a lack of analysis of the impact on firms from an organizational perspective.

### Entrepreneurial Dynamic Capabilities

Entrepreneurial dynamic capabilities are proposed around the two core elements of entrepreneurial ventures, opportunity, and resources, and refer to the ability to achieve opportunity identification and opportunity development based on the coordination and allocation of resources (Corner and Wu, 2012; Cui et al., 2020). Opportunity identification capability emphasizes the identification and utilization of objective market opportunities, i.e., the allocation of enterprise resources to meet unmet needs in the market (Suddaby et al., 2015). Opportunity development capability emphasizes the creation of opportunities based on resources, i.e., the transformation of enterprise resources into products or services and the stimulation of corresponding market demand (Suddaby et al., 2015). The effect of entrepreneurial dynamic capabilities in supporting entrepreneurial firms to cope with the turbulent market environment and seek opportunities for growth has been confirmed in prior studies (Corner and Wu, 2012). Firms with entrepreneurial dynamic capabilities are more resilient and viable (Suddaby et al., 2015).

### Strategic Decision Comprehensiveness

Strategic decision comprehensiveness is a reflection of the effectiveness of strategic decision-making, and it echoes the view of Sun et al. (2020) that “planning is determined and then moving, and knowing how to stop is rewarding” (Fredrickson, 1984). Drawing on the viewpoint of Janis and Mann (1977), this paper argues that strategic decision comprehensiveness reflects the perfection of the strategic decision-making process in terms of both breadth and depth. Strategic breadth is reflected in the inclusion of as many medium and long-term planning programs of the organization and weighing different organizational goals. Strategic depth is reflected in the clarification of the implementation plans of different strategic planning programs, grasping the resource allocation requirements for achieving various strategic goals. Strategic depth is also reflected in the clarity of the implementation plan of different strategic planning programs, the resource allocation requirements to achieve various strategic objectives and the risks and benefits of each strategic program.

### Resource Orchestration Theory

Resource orchestration theory is a dynamic extension of resource-based theory, and dynamic refers to the subjective and active behavior of managers on heterogeneous resources (Sirmon et al., 2007). Therefore, the resource orchestration theory can better respond to the characteristics of entrepreneurial enterprises and highlight the key role of entrepreneurs in the construction of enterprise competitive advantages. The resource orchestration theory points out that the fundamental for an enterprise to obtain a competitive advantage lies in meeting the value needs of customers. Companies build resource portfolios, bundle resources to form capabilities, and leverage allocation capabilities to improve strategies to match customer value needs (Sirmon et al., 2007; Yu and Wang, 2021). Specifically, building a resource portfolio refers to acquiring external resources, accumulating internal resources, and stripping off non-core resources. The ability to form bundled resources refers to the ability to stabilize, enrich, and create a business. Leveraged allocation capability refers to the mobilization and allocation of various resources, and the formation of strategic deployment to identify and utilize market opportunities.

Overall, the resource orchestration theory particularly emphasizes the influence of managers’ subjective initiative on heterogeneous resources on enterprise development (Sirmon et al., 2011). The subjective and active behavior of entrepreneurs is formed based on their individual cognition. Failure learning is the change in cognition and strategy of entrepreneurs after experiencing failure situations or events, such as forming the coping logic of entrepreneurial activities and emphasizing entrepreneur cognition. This research is based on the logical framework of “cognition-behavior-performance” and points out that the process of failure learning on the sustainable development of entrepreneurship is that entrepreneurs build resource combinations based on adjusted cognition and bundle resources to form entrepreneurial dynamic capabilities or leveraged configuration capabilities It is achieved by improving strategic thoroughness, which supports the theory of resource scheduling from an empirical perspective.

## Research Hypotheses

This research is devoted to exploring the impact of entrepreneurial failure learning on the sustainable development of new ventures. Failure learning is the change in cognition and strategy of entrepreneurs after experiencing failure situations or events, such as forming the coping logic of entrepreneurial activities and emphasizing entrepreneur cognition. Ensley et al. (2006) believe that, due to the imperfect organizational structure and operation process of new ventures, the decision-making of new ventures depends on entrepreneurs and the boundary between entrepreneurs and entrepreneurial organizations are blurred. The core of resource orchestration theory lies in the fact that managers take actions on heterogeneous resources based on individual cognition to establish a company’s competitive advantage (Sirmon et al., 2011). It can be seen that the resource orchestration theory can better respond to the characteristics of startups and provide theoretical support for the key role of entrepreneurs in the development of startups. However, there is a lack of empirical research on entrepreneurship that echoes the theoretical logic of resource arrangement. Therefore, based on the resource scheduling theory and the logic of cognition-behavior-performance, we analyze the impact mechanism of entrepreneurial failure learning on the sustainable development of entrepreneurship, so as to enrich the relevant empirical research on the resource scheduling theory. The resource orchestration theory suggests that realizing the sustainable development of enterprises through resource orchestration needs to be promoted from two aspects: capacity cultivation and strategic deployment (Sirmon et al., 2011; Yu and Wang, 2021). We probe the effect of learning from failure on entrepreneurial dynamic capabilities and strategic decision comprehensiveness separately.

By learning from failure, entrepreneurs gain cognitive and strategic adjustments through reflecting on failure experiences. It helps entrepreneurs to clarify the key information and important aspects of matching opportunities with resources in the continuous promotion of opportunity-resource integration. Then entrepreneurial dynamic capabilities can be enhanced. Based on this, the impact of learning from failure on entrepreneurial dynamic capabilities is mainly reflected as follows.

First, learning from failure enhances the opportunity recognition ability by clarifying the internal and external cognition of entrepreneurs. According to the resource orchestration theory, a company’s competitive advantage comes from the manager’s resource management based on a heterogeneous combination of resources to match the value demand in the market (Eggers and Song, 2015). Thus, the construction, bundling, and utilization of heterogeneous resources must be based on the synthesis of internal and external information and the formation of comprehensive knowledge of the resource and market contexts. Politis (2010) states that learning from failure brings unique tacit knowledge to the firm and urges entrepreneurs to update internal knowledge and acquire external knowledge. As a result, learning from failure enables entrepreneurs to gain effective insight into resource characteristics and market opportunities and then to identify resource positions that are beneficial to the construction of competitive advantage by combining the entrepreneurial firm’s resource endowment and matching its resources with market opportunities (Zhang, 2011). This helps to enhance the entrepreneurs’ entrepreneurial alertness and opportunity sensitivity and improve their opportunity recognition ability (Cope, 2011).

Second, learning from failure stimulates entrepreneurs to creatively develop opportunities. Cope (2011) points out that learning from failure is a double-loop learning process. Entrepreneurs can reflect on the underlying logic behind failure, uncover the causal relationships between dominant values, action strategies, and outcomes (Cope, 2011). Also, they can examine established values and mental models in order to rethink the logic of value creation (Cope, 2011). As a result, learning from failure can support entrepreneurs in targeting based on organizational goals and market positioning. Then, they can achieve innovative transformation of resources into products or services and enhance opportunity exploitation capabilities based on organizational resources for value extraction. Accordingly, we hypothesize:

*H1*: Learning from failure positively affects entrepreneurial dynamic capabilities.

According to the resource orchestration theory, learning from failure, which includes cognitive revision and activity improvement, enriches entrepreneurs’ cognitive information and then supports to consummate strategies (Sirmon et al., 2011).

First, cognitive revision helps entrepreneurs to accurately perceive the strategical information about the value of resources and clarify their positioning. Learning from failures helps entrepreneurs to perceive cognitive dissonance and discover that past strategies or behaviors that they thought were right were actually wrong. Therefore, prompting them to adjust their perceptions and revise their values. According to the resource orchestration theory, the construction of a unique resource portfolio is the starting point for a company to build a competitive advantage (Sirmon et al., 2011). Entrepreneurial activity advances in the process of specifying, acquiring, allocating, and utilizing resources (Corner and Wu, 2012). Therefore, learning from failure helps entrepreneurs to adjust resource value judgments in the context of the business environment and enterprise characteristics (Wiklund and Shepherd, 2003). Furthermore, entrepreneurs update resource positioning and opportunity perception, in order to clarify the heterogeneous resources of the enterprise according to the opportunity value of different resources (Wiklund and Shepherd, 2003). As a result, they can effectively support subsequent entrepreneurial behaviors and achieve enterprise competitive advantage construction (Wiklund and Shepherd, 2003).

Secondly, activity improvement helps entrepreneurs to adjust strategical actions of acquire key resources and make them successfully match with market opportunities. Rethinking failure helps entrepreneurs to develop a keen awareness of the business environment and motivates them to clarify the realization of resource reconfiguration and the allocation strategy of resource utilization. Also, activity improvement can support the firm to acquire and allocate resources to identify and exploit market opportunities for value creation and performance improvement (Rae and Carswell, 2001; Carmeli and Schaubroeck, 2008). Then entrepreneurs are encouraged to make strategical actions motivates to and creatively transform their resources them into products or services (Shepherd et al., 2019). Accordingly, we hypothesize:

*H2*: Learning from failure positively affects strategic decision comprehensiveness.

The entrepreneurial dynamic ability helps to establish the competitive advantage of the enterprise in the continuous matching of resources and market opportunities. Specifically, the opportunity recognition ability supports the competitive advantage of the enterprise. Secondly, repeatedly identifying and utilizing the objective opportunities in the external environment can enrich the experience accumulation and intellectual resources of entrepreneurs (Schildt et al., 2012). These resources are beneficial to promote the technological innovation and knowledge iteration of new ventures, prompting them to carry out innovative opportunity identification and utilization, and constructing their competitive advantages (Schildt et al., 2012).

The impact of opportunity development capability on the competitive advantage of enterprises is reflected in the following. First, it emphasizes the active market-shaping of core resources, such as technology and knowledge in existing new ventures, realizes the creative transformation of entrepreneurial resources into products or services, and provides the source power for the cultivation of competitive advantage of new ventures (Lin and Wu, 2014). Second, the play of opportunity development capability is beneficial to the cultivation of the pioneering spirit and the accumulation of market development experience of entrepreneurs (Kanf and Na, 2020). The spirit and experience will be precipitated in the form of entrepreneurial spirit or organizational culture, which will have a long-term impact on the development of subsequent new ventures and the advancement of entrepreneurial practices (Ucbasaran et al., 2009). Also, it can help them to acquire a dominant market position (Ucbasaran et al., 2009). Accordingly, we hypothesize:

*H3*: Entrepreneurial dynamic capabilities positively affect new ventures’ sustainable development.

Kraatz and Zajac (2001) argued that strategic decision comprehensiveness and strategic flexibility are especially critical when firms engage in high-risk activities. Yet new ventures often face both technological and market risks, so strategic decision comprehensiveness is critical to the survival of new ventures (Kraatz and Zajac, 2001). Based on the resource orchestration theory, the competitive advantage of enterprises comes from the dynamic acquisition and utilization of resources. The impact of strategic decision comprehensiveness on the competitive advantage of new ventures is mainly reflected in the following. First, the more thorough strategic decision-making of new ventures means that entrepreneurs have a deep understanding and precise grasp of the technological and market environment (Carmeli, 2007). In turn, it helps new ventures to pay close attention to market trends, face environmental changes calmly, prevent and resolve risks (Rhaiem and Amara, 2019). Second, a well-defined strategic decision also means that the entrepreneur has a clear understanding of the interaction between market dynamics and business planning (Zuzul and Tripsas, 2019). This is valuable for new ventures that are already facing resource shortages and other weaknesses of new ventures. And, it is useful for guiding new ventures to integrate resources and develop products in a targeted manner to establish their competitive advantages (Zuzul and Tripsas, 2019). Accordingly, we hypothesize:

*H4*: Strategic decision comprehensiveness positively affects new ventures’ sustainable development.

Comprehensively consider hypotheses 1–4, we put forward the mediation hypothesis of entrepreneurial dynamic capabilities, strategic decision comprehensiveness:

*H5*: Entrepreneurial dynamic capabilities play a mediating role between learning from failure and new ventures’ sustainable development.

*H6*: Strategic decision comprehensiveness play a mediating role between learning from failure and new ventures’ sustainable development.

**Figure 1:**
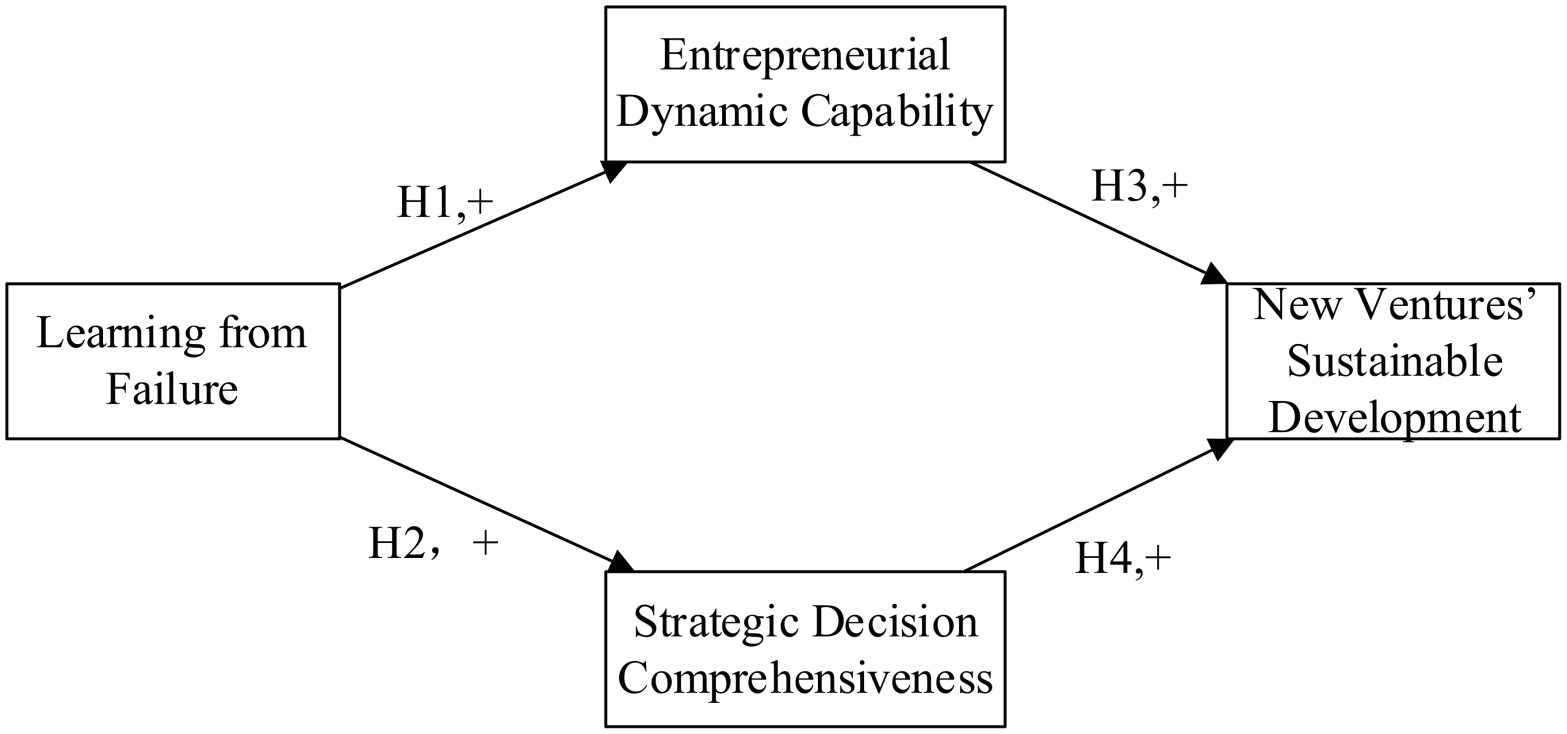
Conceptual model.

The theoretical model of this article is shown in Figure 1.

## Research Design

### Sample Selection and Data Collection

According to Cardon and Kirk (2015), this study focuses on the founders or co-founders of new ventures and uses a questionnaire survey method to collect data from them to test the conceptual model and hypotheses in order to pursue the generalizability of the findings. The data source of this study were mainly from May to September 2019, in the eastern entrepreneurial region of Mainland China, which enjoyed relatively high financial support, with the advantages of a sufficient supply of raw materials and many engineering universities nearby. Referring to the definition of new ventures by Covin and Slevin (2009) and McDougall and Robinson (2010), a new venture is an enterprise established for 8years or less. Entrepreneurs were asked to complete the questionnaire.

Entrepreneurial failure in this paper is a scenario in which the practical outcome of the entrepreneurial process deviates from the expected goal, such as the failure of financing for science and technology new ventures and the failure of new product development (Politis and Gabrielsson, 2009). The survey samples in this article come from entrepreneurs in normally surviving entrepreneurial enterprises. During the survey, we first introduce the meaning of entrepreneurial failure in the questionnaire and then ask the entrepreneur to recall whether he has experienced more than two failures. And, if he has experienced it, he can continue to complete and submit the questionnaire. We mainly look for entrepreneurs in the co-creation space. In a first way, we took a paper questionnaire and some small souvenirs to visit the entrepreneurs in the co-creation space one by one. In order not to delay the work of entrepreneurs, we chose to visit during lunch break. We communicate with entrepreneurs, listen to entrepreneurs tell their entrepreneurial stories, and ask them to fill out the questionnaire. Sometimes, we can retrieve the questionnaire from the entrepreneur on the spot, and sometimes we will retrieve the questionnaire at the time of the entrepreneur’s request. In a second way, we will contact the management department of the co-creation space. They have a WeChat group that includes all entrepreneurs in the space. Upon our request, they will send the questionnaire link to the group, so that we can retrieve the electronic version of the questionnaire.

We used three methods to minimize general method deviation. The first was by implementing anonymous filling methods to reduce the responsibilities of the person filling the questionnaire. The second was to avoid the questionnaire being filled out multiple times by the same person (filling in online and offline questionnaires at once). The third, we required the same new ventures to collect only one sample to avoid data from comparing the same company subjects. The research finally collected 232 questionnaires, and 193 valid questionnaires were obtained, with a recovery efficiency of 83.2%. Among the entrepreneurs, 33.7% were female and 66.3% were male; in terms of educational background, 8.3% were college students and below, 72.5% were undergraduates, 17.1% were master students and 2.1% were doctoral students. The participants were mainly 20–30 and 30–40years old, accounting for 48.7 and 47.2%, respectively, followed by 40–50years old (4.1%). As for the number of times of entrepreneurship, 57.5% for the first time and 42.5% for more than two times. In terms of establishment time, the sample companies are relatively young, with 55.4% of companies established within 3years, and only 3.6% of companies established over 6years. In terms of the number of employees, companies with 30 employees or less accounted for 67.9%. In terms of sales revenue, 77.2% of the sample companies with sales revenue of fewer than 5 million yuan. From the perspective of industrial distribution, the electronic information industry accounted for 21.4%, and new energy and new material industry accounted for 25.9%, the biomedicine industry accounted for 19.6, and 23.1% of the companies were located in other industries.

### Instruments

For the adopted foreign scales, some of the authors first translate the survey items, if originally in English, into Chinese and use back-translation to test accuracy (Craig and Douglas, 2006). We invite two Ph.D. candidates, who major in psychology and management with the experience of studying in English speaking countries for more than 2years, to translate them from English to Chinese. We review the original and back-translated versions to ensure they are equivalent. The questionnaire mainly includes background information of entrepreneurs or new ventures and measurement of variables. The background information of entrepreneurs or new ventures such as age, education background, firm size is measured using the form of selection or filling in the blanks. The questionnaire measurement of variables including learning from failure, entrepreneurial dynamic ability, strategic decision comprehensiveness, and new ventures’ sustainable development in this study utilizes a seven-point Likert scale with 1 indicating complete disagreement and 7 indicating complete compliance.

#### Learning From Failure

According to Cannon and Edmondson (2010) and Cope (2011), we use seven items scale, such as “learn to stop and reflect on the work process,” “clearer understanding of the company’s future development direction,” “a clearer understanding of the advantages and disadvantages of the company,” etc. The internal consistency Cronbach’s alpha coefficient was 0.62.

#### Entrepreneurial Dynamic Ability

Based on the study of Corner and Wu (2012) and Laaksonen and Peltoniemi (2018), the scale contains six items. Using the research results of Rong et al. (2011), with three items measuring entrepreneurial opportunity identification, such as “entrepreneurial sensitivity to new opportunities,” “spend more time and energy to find products and services that can bring value to consumers,” and “ability to continuously observe the market, monitor customers and competitors, and allocate resources based on market activities.” Using the research results of Foss et al. (2013), which measured entrepreneurial opportunity development ability with three items, such as “ability to develop new market areas,” “develop many new products or new services,” and “make substantial changes to the current product or service portfolio.” The internal consistency of the Cronbach’s alpha coefficient was 0.68.

#### Strategic Decision Comprehensiveness

Learning from the study of and Eggers and Song (2015), we use a scale contained five items, such as “in order to achieve the goal, several alternative action plans are usually formulated instead of one,” “usually develop multiple alternative courses of action to achieve goals,” “clarify whether some changes in the external environment are opportunities or threats,” “examine the execution process of the action plan from multiple angles,” and “will extensively collect alternative actions.” The internal consistency of the Cronbach’s alpha coefficient was 0.64.

#### New Ventures’ Sustainable Development

According to Roudini and Osman (2012), the scale contains six items, such as “the firm can provide products or services to customers at a lower cost,” “companies can provide customers with multi-functional, high-performance products or services,” “companies can execute operational procedures in a faster and more effective way,” “companies can flexibly adapt to rapidly changing markets and react faster than their opponents,” “companies pay more attention to customer needs,” and “the company’s market share is growing faster.” The internal consistency of the Cronbach’s alpha coefficient was 0.66.

#### Control Variables

With reference to existing research methods, gender, age, educational background, previous experience, firm age, and firm size is used as a control variable.

Gilford (1954) and Chinese scholar Wu (2010) both pointed out that Cronbach’s alpha coefficient greater than 0.6 is acceptable. Therefore, we carry out the following stepwise regression analysis based on this.

## Empirical Analysis and Results

### Convergent Validity

The average variance extracted (AVE) showed the degree of correlation between the construct and its indices, with a greater fit achieved with stronger correlation. Any composite-reliability (CR) rating higher than 0.7 suggests that e construct was internally acceptable (Chin, 1998). In this study, the AVE of all variables was higher than 0.5 and the CR of all variables was higher than 0.7 (Table 1).

**Table 1:** Indicators of measurement.

Constructs	Items	Factor loading	Average variance extracted (AVE)	Composite reliability (CR)
Learning from failure	LF1	0.739	0.533	0.886
	LF2	0.797		
	LF3	0.805		
	LF4	0.856		
	LF5	0.628		
	LF6	0.504		
	LF7	0.722		
Entrepreneurial dynamic ability	EDA1	0.636	0.566	0.883
	EDA2	0.617		
	EDA3	0.777		
	EDA4	0.625		
	EDA5	0.914		
	EDA6	0.883		
Strategic decision comprehensiveness	SDC1	0.726	0.535	0.846
	SDC2	0.798		
	SDC3	0.952		
	SDC4	0.572		
	SDC5	0.526		
New ventures’ sustainable development	SD1	0.885	0.520	0.860
	SD2	0.763		
	SD3	0.835		
	SD4	0.458		
	SD5	0.790		
	SD6	0.475		

### Discriminant Validity

Using SPSS22.0 and AMOS22.0 to test discriminant validity of the variables involved, we conducted confirmatory factor analysis of learning from failure, entrepreneurial dynamic capability, strategic decision comprehensiveness, and new ventures’ sustainable development. The results of the AMOS confirmatory factor analysis are then presented in Table 2. The data fit of the four-factor model (*χ*^2^/*df*=1.06, RMSEA=0.04; SRMR=0.06; CFI=0.92; TLI=0.90) was the most ideal, which was significantly better than that of the other models. Results showed that the four variables involved in this study had good discriminant validity.

**Table 2:** Confirmatory factor analysis results.

Models	*χ* ^2^	*df*	*χ*^2^/*df*	RMSEA	SRMR	CFI	TLI
Four factors	164.38	155	1.06	0.04	0.06	0.92	0.90
Three factors^a^	169.69	158	1.07	0.04	0.07	0.90	0.88
Three factors^b^	175.35	159	1.10	0.05	0.07	0.86	0.83
Two factors^c^	185.07	161	1.15	0.06	0.08	0.81	0.77
One factor^d^	188.48	162	1.16	0.06	0.08	0.80	0.76

a *Learning from Failure+Entrepreneurial Dynamic Capability, Strategic Decision Comprehensiveness, New Ventures’ Sustainable Development*.

b *Learning from Failure+Strategic Decision Comprehensiveness, Entrepreneurial Dynamic Capability, New Ventures’ Sustainable Development*.

c *Learning from Failure+Strategic Decision Comprehensiveness+Entrepreneurial Dynamic Capability, New Ventures’ Sustainable Development*.

d *Learning from Failure+Strategic Decision Comprehensiveness+Entrepreneurial Dynamic Capability+New Ventures’ Sustainable Development*.

### Descriptive Statistical Analysis

Descriptive statistics mainly display the average value, standard deviation, and correlation coefficient of each variable (as shown in Table 3). This article takes the entrepreneur’s gender, age, educational background, previous experience, firm age, and firm size as control variables. According to the results of correlation analysis, learning from failure is significantly correlated with entrepreneurial dynamic capability (*r*=0.63, *p*<0.01) and strategic decision comprehensiveness (*r*=0.56, *p*<0.01). Entrepreneurial dynamic capability (*r*=0.58, *p*<0.01) and strategic decision comprehensiveness (*r*=0.63, *p*<0.01) is significantly correlated with new ventures’ sustainable development. This provides some support for the subsequent hypothesis arguments.

**Table 3:** Descriptive statistical analysis.

	Mean	SD	1	2	3	4	5	6	7	8	9
Gender	1.34	0.47									
Age	2.55	0.57	0.06								
Educational background	4.10	0.67	0.13	0.10							
Previous experience	6.27	4.24	−0.01	0.52^**^	0.07						
Firm age	3.67	1.97	−0.00	0.17^*^	0.08	0.18^*^					
Firm size	3.58	1.64	0.17^*^	0.11	0.22^**^	0.16^*^	0.40^**^				
Learning from failure	5.11	0.76	0.01	0.08	−0.09	0.08	−0.03	0.10			
Entrepreneurial dynamic capability	5.09	0.77	−0.01	0.09	−0.10	0.08	0.00	0.13	0.63^**^		
Strategic decision comprehensiveness	5.25	0.81	−0.03	0.03	0.05	0.05	−0.10	0.11	0.56^**^	0.54^**^	
New ventures’ sustainable development	5.09	0.78	−0.01	0.10	−0.13	0.09	−0.07	0.08	0.64^**^	0.58^**^	0.63^**^

* *Significantly correlated at the 0.05 level (bilateral)*.

** *Significantly correlated at the 0.01 level (bilateral)*.

### Common Method Bias

To test the common method bias, we used Harman’s single factor test to perform an unrotated factor analysis on all collected questionnaire item data. The variance explained by the first principal component is 20.87%. This does not constitute half of the variance explained by the total variable (59.99%). Therefore, the common method bias of the sample data was within an acceptable range.

### Hypothesis Testing

Using SPSS22.0 software, hypothesis testing was performed after controlling for gender, age, educational background, previous experience, firm age, and firm size. Table 4 present the output results of the statistical analysis.

**Table 4:** Regression analysis of direct effect.

Variables	Model 1	Model 2	Model 3	Model 4
	**Outcome Variable:** Entrepreneurial dynamic capability		**Outcome Variable:** Strategic Decision comprehensiveness	
Gender	−0.03 (−0.42)	−0.03 (−0.55)	−0.06 (−0.85)	−0.06 (−1.04)
Age	0.09 (1.02)	0.05 (0.71)	0.02 (0.20)	−0.02 (−0.26)
Educational background	−0.14 (−1.83)	−0.06 (0.99)	0.03 (0.45)	0.10 (1.66)
Previous experience	0.03 (0.29)	−0.01 (−0.10)	0.04 (0.51)	0.02 (0.22)
Firm age	−0.07 (−0.94)	−0.01 (−0.19)	−0.19 (−2.34)	−0.13 (−1.96)
Firm size	0.18 (2.19)	0.09 (1.33)	0.18 (2.20)	0.10 (1.41)
Learning from failure		0.62^***^ (11.10)		0.56^***^ (9.27)
Entrepreneurial dynamic capability				
Strategic decision comprehensiveness				
VIF maximum	1.41	1.41	1.41	1.41
*R* square	0.05	0.43	0.04	0.35
Δ*R* square	0.05	0.38	0.04	0.31
*F*	1.477	19.70^***^	1.44	14.09^***^

*N=193*.

*** *Indicates p<0.001*.

** *Indicates p<0.01*.

* *Indicates p<0.05*.

In direct effects, we test the effect of learning from failure on entrepreneurial dynamic capability and strategic decision comprehensiveness, in Table 4.

In H1 and H2, we posit that learning from failure is positively related to entrepreneurial dynamic capability (H1) and strategic decision comprehensiveness (H3). As shown in Table 4, the influence coefficient of learning from failure on entrepreneurial dynamic capability (*β*=0.62, *t*=11.10, *p*<0.001) and on strategic decision comprehensiveness (*β*=0.56, *t*=9.27, *p*<0.001) were all significantly positive. Therefore, H1, H2 are supported. Specifically, when entrepreneurs can learn from failure in entrepreneurial process, the entrepreneurial dynamic capability is better. Also, when entrepreneurs can learn from failure in entrepreneurial practice, the strategic decision comprehensiveness is better.

As shown in Table 5, we test the direct and mediation effect of entrepreneurial dynamic capability and strategic decision comprehensiveness on new ventures’ sustainable development. For direct effect, in H3 and H4, we propose that entrepreneurial dynamic capability (H3) and strategic decision comprehensiveness (H4) are positively related to new ventures’ sustainable development. The results of Table 5 show that both of entrepreneurial dynamic capability (*β*=0.55, *t*=9.14 *p*<0.001) and strategic decision comprehensiveness (*β*=0.63, *t*=11.17, *p*<0.001) play positive effect on new ventures’ sustainable development. Therefore, H3, H4 are supported.

**Table 5:** Regression analysis of mediation effect.

Variables	Model 5	Model 6	Model 7	Model 8	Model 9
	Outcome variable: New ventures’ sustainable development				
Gender	−0.03 (−0.35)	−0.01 (−0.14)	−0.02 (−0.34)	0.01 (0.24)	−0.00 (−0.03)
Age	0.09 (1.13)	0.05 (0.68)	0.04 (0.70)	0.09 (1.30)	0.06 (1.08)
Educational background	−0.16 (−2.22)	−0.09 (−1.42)	−0.07 (−1.26)	−0.18 (−3.23)	−0.13 (−2.41)
Previous experience	0.05 (0.54)	0.03 (0.46)	0.02 (0.25)	0.02 (0.28)	0.01 (0.14)
Firm age	−0.14 (−1.79)	−0.10 (−1.53)	−0.08 (−1.28)	0.03 (−0.40)	−0.03 (−0.48)
Firm size	0.16 (1.99)	0.06 (0.91)	0.05 (0.74)	0.05 (0.75)	0.03 (0.50)
Learning from failure			0.48^***^ (6.91)		0.42^***^ (6.82)
Entrepreneurial dynamic capability		0.55^***^ (9.14)	0.25^***^ (3.56)		
Strategic decision comprehensiveness				0.63^***^ (11.17)	0.39^***^ (6.47)
VIF maximum	1.41	1.41	1.74	1.41	1.53
*R* square	0.06	0.35	0.48	0.44	0.49
Δ*R* square	0.06	0.29	0.13	0.38	0.05
*F*	1.882	14.26^***^	21.61^***^	20.56^***^	28.25^***^

*N=193*.

*** *Indicates p<0.001*.

** *Indicates p<0.01*.

* *Indicates p<0.05*.

From the perspective of the mediation effect, we assume that entrepreneurial dynamic capability (H5) and strategic decision comprehensiveness (H6) play a mediating role between learning from failure and new ventures’ sustainable development. As shown in Table 5, the influence coefficient of learning from failure on new ventures’ sustainable development through entrepreneurial dynamic capability (*β*=0.25, *t*=3.56, *p*<0.001) was significantly positive. The influence coefficient of learning from failure on new ventures’ sustainable development through strategic decision comprehensiveness (*β*=0.39, *t*=6.47, *p*<0.001) was significantly positive. Therefore, H5 and H6 are supported.

## Conclusion and Discussion

### Main Research Conclusion

Based on resource orchestration theory, this study explores the impact of learning from failure on new ventures’ sustainable development and the mediating role of entrepreneurial dynamic capabilities and strategic decision comprehensiveness. The main conclusions are obtained as follows.

First, learning from failure has a positive effect on entrepreneurial dynamic capabilities and strategic decision comprehensiveness, see H1 and H2. This echoes the findings of Zollo and Winter (2002) in their study that entrepreneurial learning has a positive effect on firms’ development through practice renewal. Learning from failure promotes the integration of entrepreneurial knowledge and action at both cognitive and strategic levels. It provides clear guidance for new ventures to form entrepreneurial dynamic capabilities with the essence of selecting and accessing entrepreneurial resources and identifying and developing entrepreneurial opportunities. Also, it motivates new ventures to make resource positioning and opportunity judgments by combining their resource endowments. Besides, it provides clear guidance for entrepreneurial companies to evolve strategic decision comprehensiveness in selecting and using entrepreneurial resources and identifying and developing the operation practice of entrepreneurial opportunities.

Secondly, learning from failure has a positive effect on new ventures’ sustainable development, and entrepreneurial dynamic capabilities and strategic decision comprehensiveness play mediating roles in the process, see H3, H4, H5, and H6. Entrepreneurial dynamic capabilities and strategic decision comprehensiveness can support new ventures to respond flexibly to internal and external changes and realize sustainable development. On the one hand, it echoes the findings of Kleinbaum and Stuart (2014) in their study that dynamic capabilities improve a firm’s competitive advantage. Resources and opportunities are always the core elements for new ventures to cope with changes in the economic situation and seek survival and development. On the other hand, it validates the positive effect of strategic decision circumspection on the establishment of firms’ development (Marchi et al., 2013). The findings illustrate from a resource orchestration perspective that learning from failure is an effective way for new ventures to enhance their competitive advantage in a radically changing market environment. Learning from failure implies that entrepreneurs identify cognitive biases from past setbacks or failures, achieve resource and opportunity cognitive revision to support their reconfiguration of resource portfolios. Then, they build entrepreneurial dynamic capabilities based on this by bundling resources and adjusting corporate strategies.

### Theoretical Implications

This study makes two contributions to resource orchestration research and the impact of learning from failure on new ventures development research in particular.

First, we support the logic of resource orchestration theory from an empirical view and enrich the research based on the resource orchestration theory. Resource orchestration theory states that firms establish a competitive advantage through three management behaviors including constructing resource portfolios, bundling resources to form capabilities, and leveraging and allocating capabilities to improve strategy (Sirmon et al., 2011). Based on the resource orchestration theory, with a logical framework of cognition-behavior-performance, this study points out that the impact of failure learning on the sustainable development of entrepreneurship is that entrepreneurs build resource portfolios based on adjusted cognition and bundle resources to form entrepreneurial dynamic capabilities or leveraged configuration capabilities to improve strategic decision comprehensiveness. Our study validates the theoretical model with empirical data and achieves the fit between entrepreneurial practice and resource orchestration theory.

Second, this study expands the impact of failure learning and deepens the entrepreneurship research under the guidance of the integration of opportunity and resources. This research shows that entrepreneurial dynamic capabilities and strategic decision comprehensiveness play an intermediary role in the relationship between failure learning and new ventures’ sustainable development. This emphasizes the influence of entrepreneurs on the development of entrepreneurial enterprises. It echoes the view of Ensley et al. (2006) of the difference between entrepreneurial enterprises and mature enterprises, that the boundary between entrepreneurs and entrepreneurial organizations is blurred, which is embodied in the imperfect organizational structure and operation process of entrepreneurial enterprises and corporate decision-making depends on entrepreneurship. The core leading role of entrepreneurs is obvious differences between new ventures and incumbent companies which is exactly the gist of resource orchestration theory. The conceptual model focusing on constructing new ventures’ sustainable development through entrepreneurial dynamic capabilities and strategic decision comprehensiveness embodied in entrepreneurial cognition, is conducive to the formation of a theoretical system in the field of entrepreneurship.

### Practical Implications

For entrepreneurs who hope to obtain new ventures’ sustainable development, they must first clarify the importance of learning from failure. In the face of a complex and changing business environment, entrepreneurs should focus on failure experiences, discover the value of failure through learning behaviors and develop positive interpretations of failure scenarios such as technology development or new product development. They can pay attention to both the positive effects of learning from failure and the ways to achieve positive effects through learning from failure, in order to develop a failure or crisis coping mechanism, such as regularly reporting about failure and brainstorming about how to deal with the failure. Furthermore, the entrepreneur is better to cultivate failure learning organizational culture including reviewing and learning from the failure event to develop improvement ability. There is often a “success bias” in the entrepreneurial process, where the topic of failure is avoided because of the fear of failure. However, it is often said that “failure is a common occurrence in the military” and “failure is the mother of success.” As the saying goes, the market is like a battlefield, and enterprises, especially new ventures, often suffer from internal and external attacks, internal organizational problems. The dual pressure makes the new ventures struggle or even declares bankruptcy. The uncertain environment is a serious test for companies, e.g., the ongoing epidemic around the world or the entry of new competitors. Although they face the same difficulty, different companies can come up with different ways to deal with it and reap different results accordingly. This difference comes down to the crisis management and resilience of enterprises, which have been honed through the trials and failures they have faced time and again.

Secondly, entrepreneurs who hope to obtain new ventures’ sustainable development, also need to realize the importance of entrepreneurial dynamic capabilities. Combined with the findings of this study, new ventures are able to improve entrepreneurial dynamic competencies and strategic decision comprehensiveness through failure learning and support the firm to realize sustainable development. Therefore, entrepreneurs must give effort to the reflection and summary of failure experience, analyze the causes of failure with the help of thinking such as review and summary, in order to revise and enrich the cognition of the organization itself and the external environment. Further, a keen sense of market risk identification and entrepreneurial opportunity acquisition can be built, then the standardized crisis response mechanism. In this way, it is possible for entrepreneurs to constantly seek new dynamic development windows, seize the market timing, or independent development of market opportunities to obtain a market advantage and resolve the risk to achieve a turnaround.

Last but not least, entrepreneurs are encouraged to improve strategic decision comprehensiveness to obtain new ventures’ sustainable development. As Military Science of Sun Zi says, “Knowing the enemy and yourself, you can fight a hundred battles and win them all.” In the context of economic globalization, entrepreneurs should lead the new ventures to seek competitive advantages based on a comprehensive and systematic understanding of the business environment, as well as the dual entrepreneurial spirit of active defense and active offense. It is necessary to focus on strategic decision-making to be “prepared” and to emphasize the dynamic ability of entrepreneurship to “forge ahead.” Specifically, an entrepreneur should prepare multiple sets of entrepreneurial practices based on comprehensive consideration of entrepreneurial resources and the business environment. Then, they need to combine past failure experiences to avoid failure triggers and deviation of subsequent entrepreneurial practices from organizational goals and to achieve “preparedness.” Entrepreneurs are encouraged to gain insight into business dynamics, identify market demand, and then develop new products to respond to the demand with the help of resources at hand or adopt resource piecing behavior to ride the wind. Or they can do the opposite, construct value acquisition logic based on the resource endowment characteristics of the enterprise, and break the wave with the help of business model innovation, etc. to realize the “pioneering” in entrepreneurship.

### Shortcomings and Prospects

First, the measurements of the variables in this study are mostly derived from direct adaptations of foreign scales, lacking scientific validation of the scales in the Chinese context. This may result in a biased reflection of Chinese entrepreneurial practices and make it difficult to achieve a true measure of the Chinese entrepreneurial context. Second, learning from failure is the behavior after goal deviation in entrepreneurship, which includes processes such as experience reflection, cognitive revision, and strategy adjustment, etc. Using only cross-sectional data to analyze the impact of learning from failure may lead to the problem of taking data out of context and weaken the logic of relationship construction among variables. Third, the samples in this study were mainly from incubators such as co-creation spaces, which reduced the difficulty of sample collection but at the same time limited the external validity of this study. And, future studies could select a wide range of sample companies to further validate the findings of this study. Besides, this study adopted the same questionnaire to investigate each variable, which is prone to common method bias, but the results of the Harman one-way test in this study indicate that the common method bias in this study is not significant. It should also be noted that the internal consistency of the scales, between 0.6 and 0.7, is relatively low. This may be related to the fact that we concentrated on collecting questionnaires during the lunch break of entrepreneurs, which affected the quality of filling out.

Future research can conduct a more reasonable program design to address the above issues, such as developing a learning from failure scale based on the Chinese entrepreneurial context. The continuing study can use time series data to track the learning from failure process of entrepreneurs, in order to explore the process of learning from failure and its mechanism of influencing entrepreneurial practice. By choosing different channels to enrich the research data with the help of triangulated validation thinking in case studies also a future orientation to effectively avoid the influence of common method bias on the data, and corroborate the research findings from multiple perspectives. In addition, future research can deepen both the antecedents and consequences of learning from failure in order to explore the ways and meanings of motivating or guiding entrepreneurs to carry out learning from failure and achieve sustainable entrepreneurial growth.

## Data Availability Statement

The raw data supporting the conclusions of this article will be made available by the authors, without undue reservation.

## Author Contributions

ES collected the data. ES and XZ wrote the first draft. XZ and LZ analyzed the data and contributed to research problem formulation, theory, analysis, and conclusion. All authors contributed to the article and approved the submitted version.

## Funding

This research was funded by the Shandong Social Science Planning Project (19BJCJ09).

## Conflict of Interest

The authors declare that the research was conducted in the absence of any commercial or financial relationships that could be construed as a potential conflict of interest.

## Publisher’s Note

All claims expressed in this article are solely those of the authors and do not necessarily represent those of their affiliated organizations, or those of the publisher, the editors and the reviewers. Any product that may be evaluated in this article, or claim that may be made by its manufacturer, is not guaranteed or endorsed by the publisher.
